# Acculturation and Subsequent Oral Health Problems Among Older Chinese Americans: The Role of Neighborhood Disorder

**DOI:** 10.1093/geroni/igaa057.2901

**Published:** 2020-12-16

**Authors:** Weiyu Mao, Bei Wu, Iris Chi, Wei Yang, XinQi Dong

**Affiliations:** 1 University of Nevada, Reno, Reno, Nevada, United States; 2 New York University, New York, New York, United States; 3 University of Southern California, Los Angeles, California, United States; 4 Rutgers University, New Brunswick, New Jersey, United States

## Abstract

To further understand social, cultural, and personal predictors of oral health outcomes, this study addressed the relationship between acculturation and subsequent oral health problems and tested the moderating role of neighborhood disorder in such a relationship among older Chinese Americans. The working sample included 2,706 foreign-born community-dwelling older Chinese Americans aged 60 years or older who participated in the Population Study of Chinese Elderly in Chicago at the baseline and the first follow-up. Stepwise Poisson regression using lagged dependent variable was conducted. Behavioral acculturation was protective against subsequent oral health problems. Residence in Chinatown was associated with an increase in the risk of subsequent oral health problems. The relationship between behavioral acculturation and subsequent oral health problems varied by levels of neighborhood disorder. To reduce oral health-related disease burdens, it is important to consider the role of acculturation and the neighborhood on subsequent oral health problems in practice and policy. Part of a symposium sponsored by the Oral Health Interest Group.

